# Predictive values of pre-treatment brain age models to rTMS effects in neurocognitive disorder with depression: Secondary analysis of a randomised sham-controlled clinical trial

**DOI:** 10.1080/19585969.2024.2373075

**Published:** 2024-07-04

**Authors:** Hanna Lu, Jing Li, Sandra Sau Man Chan, Suk Ling Ma, Vincent Chung Tong Mok, Lin Shi, Arthur Dun-Ping Mak, Linda Chiu Wa Lam

**Affiliations:** a Department of Psychiatry, The Chinese University of Hong Kong, Hong Kong SAR, China; b The Affiliated Brain Hospital of Guangzhou Medical University, Guangzhou, China; c Department of Medicine and Therapeutics, The Chinese University of Hong Kong, Hong Kong SAR, China; d Department of Imaging and Interventional Radiology, The Chinese University of Hong Kong, Hong Kong SAR, China

**Keywords:** rTMS, MRI, brain age, neurocognitive disorder, prediction, cortical features, depression, neuroplasticity

## Abstract

**Introduction:**

One major challenge in developing personalised repetitive transcranial magnetic stimulation (rTMS) is that the treatment responses exhibited high inter-individual variations. Brain morphometry might contribute to these variations. This study sought to determine whether individual’s brain morphometry could predict the rTMS responders and remitters.

**Methods:**

This was a secondary analysis of data from a randomised clinical trial that included fifty-five patients over the age of 60 with both comorbid depression and neurocognitive disorder. Based on magnetic resonance imaging scans, estimated brain age was calculated with morphometric features using a support vector machine. Brain-predicted age difference (brain-PAD) was computed as the difference between brain age and chronological age.

**Results:**

The rTMS responders and remitters had younger brain age. Every additional year of brain-PAD decreased the odds of relieving depressive symptoms by ∼25.7% in responders (Odd ratio [OR] = 0.743, *p* = .045) and by ∼39.5% in remitters (OR = 0.605, *p* = .022) in active rTMS group. Using brain-PAD score as a feature, responder-nonresponder classification accuracies of 85% (3^rd^ week) and 84% (12^th^ week), respectively were achieved.

**Conclusion:**

In elderly patients, younger brain age appears to be associated with better treatment responses to active rTMS. Pre-treatment brain age models informed by morphometry might be used as an indicator to stratify suitable patients for rTMS treatment.

**Trial registration:**

ClinicalTrials.gov Identifier: ChiCTR-IOR-16008191

## Introduction

In the past decades, neuroimage-guided repetitive transcranial magnetic stimulation (rTMS), has been experimented as a non-invasive technology to manage symptoms of a range of brain disorders (Takeuchi et al. 2005; Sayar et al. 2013; Mi et al. 2020; Hopman et al. 2021). However, individual differences in the treatment outcomes in elderly patients inferred from published observational studies and clinical trials presented considerable challenges in the interpretation of rTMS effects at the individual level. For instance, with the same number of TMS pulse, the elderly patients had a lower response rate (23%) than young patients (56%) (Figiel et al. 1998). Later, a comparable study found a higher response rate in elderly patients (45%) (Fabre et al. 2004). It seems that age plays an important role in the evaluation of treatment responses to rTMS. Interestingly, the association between advanced chronological age and clinical remission in elderly patients was also observed in our previous work (Lu et al. 2022). Herein, it should be noticed the complex mechanisms behind the ‘age-response’ relationship in elderly patients, of which the predominant one might be the neuroanatomical variances due to age-related morphometric changes (i.e., loss of brain parenchyma), rather than increased chronological age (Rutherford et al. 2017; Kirsch 2019; Taylor 2023).

With the development of quantitative approaches in neuroimaging, the concept of the brain age model has been developed for thoroughly estimating an individual’s chronological age from MRI data (Franke and Gaser 2012; Cole et al. 2019; Sanford et al. 2022; Bacas et al. 2023). Currently, the brain age model contains two components: (1) estimated brain age; (2) brain-predicted age difference (brain-PAD). The brain-PAD score is defined as the difference between estimated brain age (BA) and chronological age (CA) (Franke and Gaser 2012; Cole et al. 2020; Wrigglesworth et al. 2022), which has threefold explanations: (1) a negative score of brain-PAD representing decelerated brain ageing (i.e., BA < CA); (2) a positive score of brain-PAD representing accelerated brain ageing (i.e., BA > CA); (3) brain-PAD score equal to zero, representing normal brain ageing (i.e., BA = CA). Based on the nature of the quantitative approach, the brain age model is increasingly considered to be a useful neuroimaging marker in ageing populations and patients with neurodegenerative diseases. For instance, a positive score of brain-PAD has been reported in individuals with normal ageing (Lee et al. 2022), mild cognitive impairments (MCI) (Gaser et al. 2013), neurocognitive disorder (Lu et al. 2022) and dementia (Wang et al. 2019). Although the volume-based brain age models have been well developed in the last decade (Franke and Gaser 2012; Gaser et al. 2013; Wang et al. 2019; Lee et al. 2022; Lu et al. 2022), the accurate estimation of brain age requires computational models that capture and quantify the underlying age-related morphometric changes. Beside of volume, cortical thickness, representing the depth of cortical gray matter, is relevant to the treatment effects induced by transcranial brain stimulation (Baeken et al. 2021; Nissim et al. 2022; Lu et al. 2022). When it comes to the brain age model, volume-based and thickness-based brain-PAD showed distinct patterns in the clinical population (Zhu et al. 2023). These results indicate that the brain age models computed with different morphometric features, may yield dissimilar or even conflicting results, which can limit their utility in identifying the potential responders to transcranial brain stimulation.

To our knowledge, no published studies have investigated the individual differences in the treatment responses to rTMS and whether the pre-treatment brain age model could serve as a personalised predictor of response to rTMS. Thus, the main goal of the present study was to estimate individual’s brain age models *via* morphometric features and machine-learning models, and to assess their predictive capacities between responders and non-responders to rTMS. Considering the status of cognitive decline and depression are linked to accelerated brain ageing (Franke et al. 2010; Gaser et al. 2013; Han et al. 2021; Dintica et al. 2023), we hypothesised that: (1) the patients with comorbid depression and cognitive decline would have older estimated brain age and positive brain-PAD score; (2) pre-treatment morphometric feature-specific brain age models might have predictive values for identifying the potential rTMS responders.

## Materials and methods

### Data sources and study participants

The study was a secondary analysis of a single blind, sham-controlled randomised clinical trial (RCT) (Lu et al. 2022). The logistics of this trial followed the Consolidated Standards of Reporting Trials (CONSORT) flow diagram (Supplementary Figure S1). Briefly, one hundred participants were screened, 55 elderly patients with comorbid depression and neurocognitive disorder (NCD) were recruited and randomly assigned to receive either active rTMS or sham rTMS treatment. During the treatment, three patients withdrew from this trial because of the headache induced by rTMS.

We considered participants eligible if they satisfied the following inclusion criteria (APA, 2013): (1) Adults aged from 60 to 90 years; (2) with a DSM-5 diagnosis of either major neurocognitive disorder due to Alzheimer’s disease (NCD-AD) or neurocognitive disorder due to vascular disease (NCD-vascular), including a significant cognitive decline in the core domains of cognitive function (e.g., attention, memory, perceptual-motor, language and executive function), and with clinical or imaging features indicative of either Alzheimer’s disease or neurovascular disease; (3) with a total score of Cornell scale for depression in dementia (CSDD) equal to 7 or above, indicating a status of clinically depression (Pieper et al. 2016). Throughout the trial, anti-dementia and other psychotropic medication were kept constant.

We excluded the participants who had a history of primary bipolar or other psychotic disorders, and major neurological disorders, including stroke, transient ischaemic attack or traumatic brain injury. The participants were also excluded if they were unable to attend the magnetic resonance imaging scanning or rTMS session due to contraindications (e.g., metal on or inside the body).

### Randomisation and masking

Participants were randomly assigned 1:1 to one of two interventions: (1) active 10 Hz rTMS, (2) sham 10 Hz rTMS. In order to ensure equally allocation across treatment groups, prior to opening the study for enrolment, the randomisation assignment was generated using an online system (http://randomisation.com/) by a statistician not involved in the study design. Assessment staff and participants were blinded to group allocation.

### Treatments and study visits

#### Pre-treatment preparation

Structural magnetic resonance imaging (MRI) data was acquired using a 3.0 T MRI scanner (Philips Healthcare, Best, Netherlands) at the Prince of Wales Hospital in Hong Kong. The T1-weighted magnetisation prepared rapid gradient echo (MPRAGE) sequence was used for optimising the grey-white contrast, with the parameters as: repetition time (TR) = 7.6 ms, echo time (TE) = 3.5 ms, axial acquisition with a 256 × 256 × 192 matrix, flip angle (FA) = 15°, the field of view (FOV) = 230 mm, thickness = 0.6 mm, and the voxel size of 1 mm × 1 mm × 1 mm.

#### Image-guided rTMS treatment

**Figure 1.**
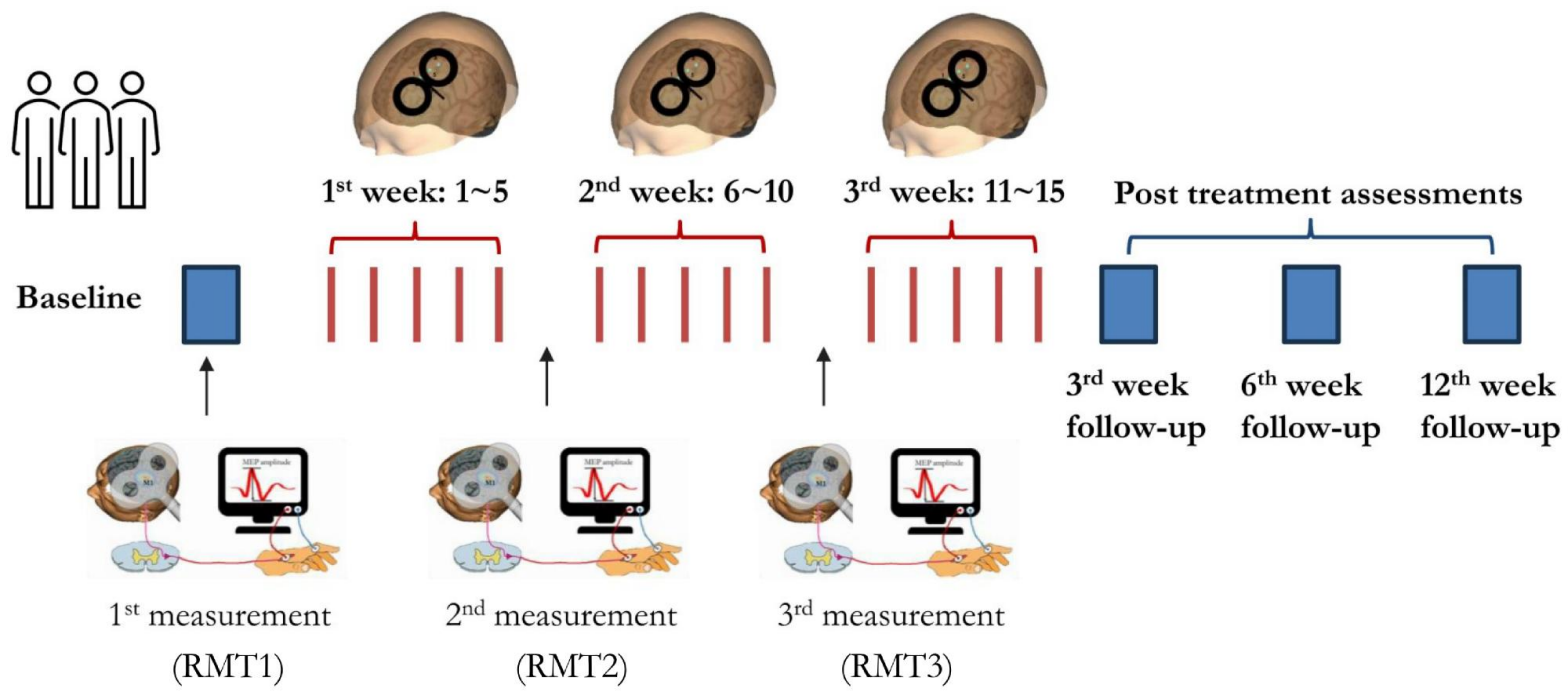
The logistics of current randomised clinical trial and neuroplastic measurements. For determining the cortical excitability at individual level, resting motor threshold (RMT) was measured at baseline (1^st^ time), after the 5^th^ session (2^nd^ time) and the 10^th^ session (3^rd^ time) of rTMS. The post-treatment outcomes were assessed at the 3^rd^ week, 6^th^ week and 12^th^ week.

The details of image-guided rTMS treatment can be found in our previous work (Lu et al. 2022). Briefly, a pre-treatment MRI-informed head model was constructed for pinpointing the treatment targets. For quantifying and monitoring the cortical excitability at the individual level, resting motor threshold (RMT) was measured at baseline (RMT1), after the 5^th^ session of rTMS (RMT2) and after the 10^th^ session of rTMS (RMT3) (Figure 1). RMT was defined as the minimum stimulus intensity of the single-pulse TMS over the left primary motor cortex (M1) that could trigger a motor evoked potential (MEP) with a peak-to-peak amplitude ≥50 μV in at least 5 out of 10 consecutive trials recorded in the electromyography (EMG) system (Fitzgerald et al. 2009).

For each participant, we pinpointed the location of the left DLPFC with the MNI coordinates as [x = –46, *y* = 45, *z* = 38], being carefully to locate this region within the grey matter on the top of the middle frontal gyrus (MFG) in the Brainsight system (Fitzgerald et al. 2009; Fox et al. 2012; Lu et al. 2019). The procedure of sham rTMS followed the same steps described above, except that a sham TMS coil was used for sham rTMS treatment.

##### Schedule of rTMS treatment

As shown in Figure 1, fifteen sessions of 10 Hz rTMS treatment were conducted by trained clinician. Outcome measures were collected at four time points, including T0 (baseline, pre-treatment), T1 (3^rd^ week, post-treatment), T2 (6^th^ week, 3 weeks post- treatment) and T3 (12^th^ week, 6 weeks post- treatment).

### Morphometric feature-based brain age models

#### Quantifications of morphometric features

**Figure 2.**
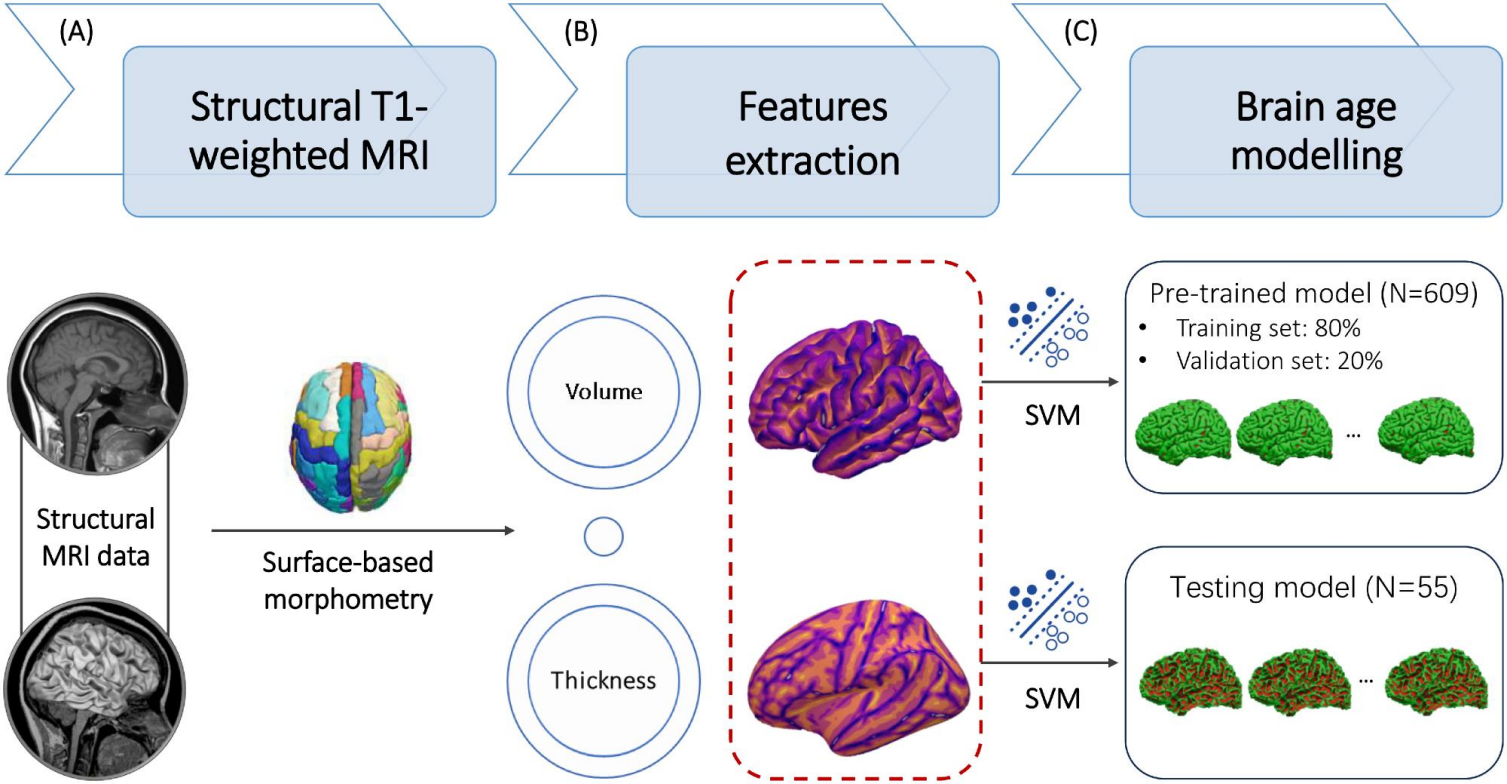
The pipeline of morphometric features mapping and brain age model calculation. Based on individual structural MRI data, the surface-based morphometry (SBM) analysis of morphometric features was performed to extract and quantify the morphometric features (A), including gray matter volume (GMV) and cortical thickness (CT) (B). (C) The estimated brain age and brain-PAD were calculated based on the quantified morphometric features using support vector machine (SVM) in the Cam-CAN dataset and our clinical samples.

Individual’s morphometric features of the brain, including gray matter volume (GMV) and cortical thickness (CT), were extracted and quantified by BrainSuite (http://brainsuite.org/). BrainSuite is an automatic cortical surface identification integrated package with the refined algorithm of brain surface extraction, which are suitable for individuals with brain atrophy (Huppertz et al. 2010; Lu et al. 2016; Gorges et al. 2020). Based on the results of tissue segmentation, the cortical surface for each participant were reconstructed by a deformable surface method (Figure 2A). The morphometric features represent the mathematical measures of the reconstructed cerebral cortex (i.e., gyrus), including GMV and CT (Figure 2B). CT is calculated as an average of the distance from the white matter surface to the closest point on the pial surface and from that point back to the closest point to the white matter surface (Vuksanović et al. 2019; Lu 2020).

#### Brain age models

In the stage of estimation, we extracted the whole brain features from the training samples derived from the Cambridge Centre for Ageing and Neuroscience (Cam-CAN) (*N* = 609, age range: 18-90 years) (https://www.cam-can.org) (Taylor et al. 2017), and then generated a machine learning-driven pre-trained brain age model. Based on an individual’s GMV and CT, estimated brain age was predicted using a support vector machine (SVM) implemented in MATLAB (‘fitrsvm’ function, kernel: linear) (Figure 2C). Using ten-fold cross-validation, the pre-trained brain age model was applied to the vectorised study images to estimate the brain age for the entire samples (*N* = 609). The dependent variable was chronological age. The independent variables encompass the GMVs of 68 cortical regions and CTs of 66 cortical regions. The total dimensionality of the independent variables is 134, and the details of the brain regions were outlined in Supplementary Table 1.

Using principal component analysis (PCA) analysis, the top principal components capturing 80% of the variance in morphometric features were retained (Biondo et al. 2022). The details of Cam-CAN samples and pre-trained brain age models can be found at Github (https://github.com/hannabrainscience/Brain-age-prediction). Participants with pre-treatment MRI scans from the current clinical trial were used as a testing set (*N* = 55) for calculating and validating the GMV-based and CT-based brain age models (Figure 2C). Considering the participants in the current clinical trial aged 66–87 years (i.e., elderly), age-bias correction was applied in the estimation of brain age (Li and Lu 2023). Three indexes of model error statistics, including bivariate correlation between estimated brain age and chronological age (*r*), mean absolute error (MAE), and root mean square error (RMSE), were used to evaluate the model performance in both pre-trained and testing brain age model (Christman et al. 2020; Cole 2020).

### Measures of treatment outcomes

#### Primary outcomes

1. Severity of depression: The Cornell Scale for Depression in Dementia (CSDD) was used for assessing depressive symptoms (Schreiner et al. 2003). In this study, a cut-off CSDD total score of seven indicated clinically significant depressive symptoms. Responders were defined as having a 50% reduction in the total score of CSDD after fifteen sessions of rTMS treatment (Lyketsos et al. 2003). Remitters were defined as having a total score of CSDD lower than seven (i.e., the cut-off score of depression) at the post-treatment time points (Lu et al. 2022).
2. Global cognitive function: The Montreal Cognitive Assessment Hong Kong version (HK MoCA) was used to measure the global cognitive function (Wong et al. 2009). HK MoCA is a 30-point test including visuospatial ability, naming, executive function, language, memory, attention, abstract and orientation, which has been validated in age-related neurodegenerative diseases (Lu et al. 2016).

#### Secondary outcomes

Cortical excitability: The resting motor threshold (RMT) induced by single pulse TMS was used as a proxy measure of cortical excitability (Pascual-Leone et al. 1998; Fitzgerald et al. 2006; Buch et al. 2011). During this trial, three times of RMT measurement were conducted at baseline (RMT1), after the 5^th^ session of rTMS (RMT2) and after the 10^th^ session of rTMS (RMT3) (Figure 1). The changes between pre-treatment and post-treatment RMT were used to evaluate the level of cortical excitability related to repeated rTMS (i.e., RMT1-RMT2, RMT1-RMT3).

### Statistical analysis

Analyses were performed by the ‘intention-to-treat’ principles. Each patient was classified as either an rTMS ‘non-responder’/‘responder’ or a ‘non-remitter’/‘remitter’ on the basis of different rating scales (i.e., a 50% reduction in the total score of CSDD for the responder, and a total score of CSDD lower than seven for the remitter) to determine the treatment outcomes at post-treatment time points. Pre-treatment clinical and whole brain morphometric features were compared between the non-responders and responders, and between the non-remitters and remitters using a two-sample *t*-test for continuous variables.

For the predictive models, we evaluated the morphometric feature-specific estimated brain age and brain-PAD in predicting the responses to rTMS treatment. Next, the potential predictor variables were included in logistic regression models to determine their associations with response, remission and the changes of cognition and cortical excitability as measured by odds ratio (OR) with the corresponding 95% confidence intervals (CI). We employed exploratory logistic regression models and receiver operating characteristic (ROC) analyses with dichotomised treatment outcomes (non-responder/responder or non-remitter/remitter) and estimated brain age and brain-PAD as predictor variables to evaluate the utility of brain age models to predict patient’s responses to rTMS treatment. All statistical analyses were conducted using SPSS Statistics 24.0 (IBM, Armonk, NY). Statistical tests were two-tailed, with the alpha (Type I error rate) set to 0.05.

## Results

### Demographic and clinical characteristics

As depicted in the CONSORT flow diagram (Figure S1), one hundred participants were screened for eligibility of this clinical trial, of which 55 patients were randomly assigned to receive active rTMS (*n* = 27) or sham rTMS (*n* = 28). The baseline demographics in terms of sex and years of education, global cognitive function and the severity of depression were comparable between the active and sham rTMS groups (Table S1).

### Responders and remitters to rTMS

As mentioned, responders were defined as the patients with a 50% reduction in the total score of CSDD after rTMS treatment. Remitters were defined as patients having a total score of CSDD lower than seven at the post-treatment time points. At the post-treatment point (T1), four responders and eight remitters were identified in the active rTMS group. The responders and remitters had a higher total score of HK MoCA than the non-responders and remitters (Table 1). Compared to the non-remitters, the remitters in the active rTMS group had higher chronological age (*t* = −3.407, *p* = .003), less severe depressive symptoms (*t* = 2.121, *p* = .045). In the sham rTMS group, four responders and three remitters were identified. As shown in Table 2, no differences of chronological age and pre-treatment clinical features were found between the non-remitters and remitters in the sham rTMS group.

**Table 1.** Pre-treatment demographic and clinical features of the responders and remitters at trial endpoint.

Clinical features	Response				Remission			
	Non-responders	Responders	*t* (*χ^2^*) value	*p* value	Non-remitters	Remitters	*t* (*χ^2^*) value	*p* value
**Active rTMS group**								
Age	68.53 ± 7.24	73.54 ± 4.25	−1.326	0.199	66.51 ± 5.57	75.07 ± 6.28	−3.407	0.003
Sex (F/M)	14/6	3/1	−0.192	0.849	12/4	5/3	0.613	0.546
Education (years)	7.15 ± 4.55	10.51 ± 3.11	−1.397	0.176	7.06 ± 4.71	9.01 ± 3.89	−1.002	0.327
HK MoCA	19.01 ± 5.32	25.5 ± 3.69	−2.313	0.031	18.63 ± 5.74	23.01 ± 4.21	−2.117	0.048
CSDD	12.55 ± 4.69	12.75 ± 4.19	−0.079	0.938	13.88 ± 4.31	10.01 ± 4.04	2.121	0.045
QoL-AD	29.27 ± 5.57	33.25 ± 6.89	−1.214	0.241	27.92 ± 5.16	33.86 ± 5.46	−2.371	0.031
**Sham rTMS group**								
Age	74.04 ± 7.63	73.73 ± 6.55	0.076	0.941	74.27 ± 7.53	71.84 ± 6.54	0.532	0.601
Sex (F/M)	17/5	1/3	2.193	0.038	17/6	1/2	1.434	0.165
Education (years)	6.82 ± 4.69	9.25 ± 8.54	−0.839	0.411	7.22 ± 4.97	7.01 ± 8.89	0.065	0.948
HK MoCA	18.73 ± 6.87	21.51 ± 2.52	−0.786	0.441	18.87 ± 6.75	21.33 ± 3.06	−0.615	0.544
CSDD	11.01 ± 3.77	12.01 ± 3.46	−0.493	0.626	11.26 ± 3.89	10.33 ± 1.16	0.404	0.691
QoL-AD	27.73 ± 8.09	25.75 ± 8.06	0.419	0.682	26.92 ± 8.21	28.33 ± 7.57	−0.271	0.791

Data are raw scores and presented as mean ± SD.

rTMS: Repetitive transcranial magnetic stimulation; HK MoCA: Montreal Cognitive Assessment Hong Kong version; CSDD: Cornell Scale for Depression in Dementia; QoL-AD: Quality of Life in Alzheimer’s disease.

**Table 2.** A summary of the prediction accuracy of morphometric feature-specific brain age models.

Cortical features	*R* ^2^	MAE (years)	RMSE (years)
Pre-trained model (*N* = 611)			
GMV-based brain age	0.71	7.82	9.71
CT-based brain age	0.76	7.03	8.94
Testing model (*N* = 55)			
GMV-based brain age	0.75	7.42	9.21
CT-based brain age	0.77	7.16	8.91

Data are raw scores and presented as mean ± SD.

MAE: Mean absolute error; RMSE: Root mean square error; GMV: Gray matter volume; CT: Cortical thickness.

### Estimated brain age and brain-PAD

The performance of brain age models was summarised in Table 2, which showed comparable generalisability with published brain age models (de Lange et al. 2022; Karim et al. 2022). Chronological age was positively correlated with estimated brain age in the training samples (*r* = 0.807, *p* < .001) and testing samples (active rTMS: *r* = 0.606, *p* = .002; sham rTMS groups: *r* = 0.522, *p* = .007) (Figure S2). The NCD patients had comparable chronological age (*t* = 2.623, *p* = .077), but higher brain age (*t* = 10.886, *p* < .001) and brain-PAD (*t* = 15.378, *p* < .001) than healthy controls in the training set (see details in Table S2). The pre-treatment brain age was comparable in the active and sham rTMS groups (*t* = −0.462, *p* = .646).

**Figure 3.**
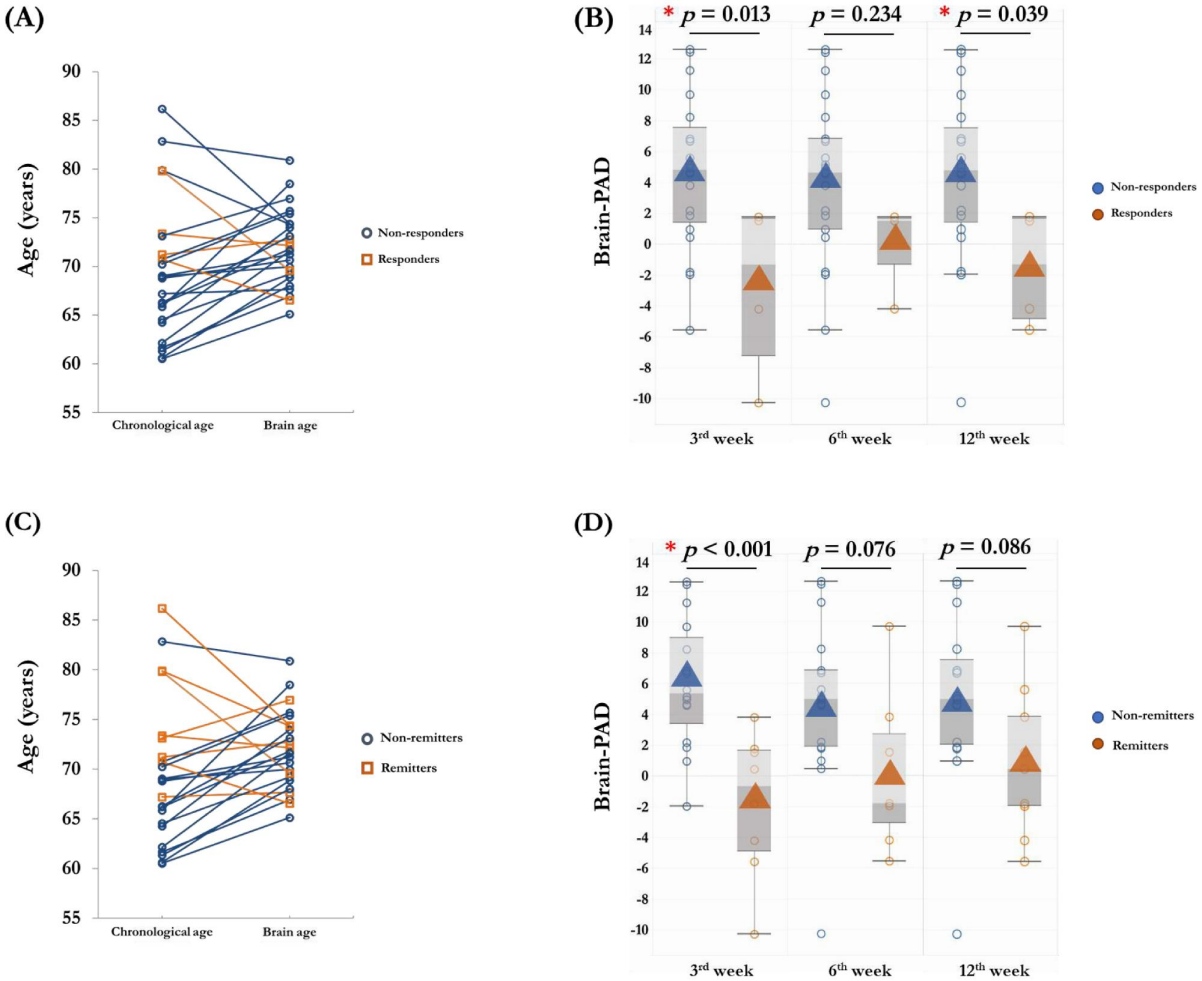
The differences of brain age models in responders and remitters in active rTMS group. At 3^rd^ week, the individual differences between chronological age and brain age were found in the responders (A) and remitters (C). Significant lower CT-based brain-PAD scores were found in the responders in active rTMS group at the 3^rd^ week (*p* = .013) and 12^th^ week (*p* = .039) (B). Significant lower CT-based brain-PAD scores were found in the remitters in active rTMS group at 3^rd^ week (*p* < .001).

As shown in Table 3, pre-treatment morphometric features, including global GMV and mean CT, and GMV-based brain age and CT-based brain age were comparable between the non-responders and responders, and between the non-remitters and remitters in the active rTMS group. At 3^rd^ week, the individual differences between chronological age and brain age were found in the responders (Figure 3A) and remitters (Figure 3C). Although similar patterns of brain-PAD were observed in the responders and remitters at the post-treatment time points, significant lower scores of CT-based brain-PAD were found in the responders at 3^rd^ week (*t* = 2.713, *p* = .013) and 12^th^ week (*t* = 2.655, *p* = .039) (Figure 3B) in active rTMS group. The remitters only showed a lower score of CT-based brain-PAD at 3^rd^ week in the active rTMS group (*t* = 4.151, *p* < .001) (Figure 3D). In the sham rTMS group, thinner cortical thickness was found in the responders (*t* = 2.969, *p* = .007) and remitters (*t* = 2.225, *p* = .036) (Table 3). No differences of pre-treatment estimated brain age, brain-PAD and other morphometric features were observed between the non-responders and responders, and between the non-remitters and remitters in the sham rTMS group.

**Table 3.** Pre-treatment morphometric features and brain age models of the responders and remitters in randomised rTMS groups.

MRI-informed brain features	Response				Remission			
	Non-responders	Responders	*t* value	*p* value	Non-remitters	Remitters	*t* value	*p* value
**Active rTMS group**								
Global GMV	416.31 ± 56.52	415.41 ± 31.04	0.031	.976	410.31 ± 53.15	427.84 ± 52.89	−0.762	.454
Global WMV	262.88 ± 41.51	207.91 ± 72.33	1.472	.227	258.64 ± 38.22	243.86 ± 71.13	0.669	.511
Mean CT	3.59 ± 0.37	3.81 ± 0.56	−0.946	.355	3.63 ± 0.36	3.62 ± 0.49	0.022	.983
GMV-based brain age	72.45 ± 4.36	71.18 ± 7.09	0.482	.635	72.03 ± 4.34	71.08 ± 5.75	0.455	.653
GMV-based brain-PAD	2.65 ± 6.82	−1.08 ± 8.14	0.974	.341	4.57 ± 4.88	−3.04 ± 8.12	2.881	.009
CT-based brain age	73.12 ± 4.72	70.76 ± 3.41	0.943	.356	72.44 ± 4.03	73.31 ± 5.73	−0.428	.673
CT-based brain-PAD	4.64 ± 4.88	−2.78 ± 5.69	2.713	.013	5.99 ± 4.16	−1.77 ± 4.66	4.151	<.001
**Sham rTMS group**								
Global GMV	409.08 ± 48.12	388.48 ± 27.88	0.822	.419	407.07 ± 47.98	396.98 ± 27.08	0.353	.727
Global WMV	240.65 ± 41.78	251.21 ± 29.45	−0.481	.635	240.11 ± 40.89	258.86 ± 30.82	−0.761	.454
Mean CT	3.81 ± 0.41	3.19 ± 0.13	2.969	.007	3.79 ± 0.43	3.22 ± 0.14	2.225	.036
GMV-based brain age	72.32 ± 5.11	72.13 ± 2.21	0.074	.941	72.44 ± 5.01	71.19 ± 1.42	0.422	.677
GMV-based brain-PAD	−1.82 ± 5.94	−1.61 ± 6.75	−0.066	.948	1.94 ± 5.82	0.64 ± 7.92	0.349	.731
CT-based brain age	74.12 ± 4.55	70.93 ± 3.21	1.332	.195	74.02 ± 4.47	70.59 ± 3.83	1.265	.218
CT-based brain-PAD	0.08 ± 6.21	−2.81 ± 8.38	0.813	.424	−0.25 ± 6.27	−1.25 ± 9.53	0.246	.807

Data are raw scores and presented as mean ± SD.

rTMS: Repetitive transcranial magnetic stimulation; Brain-PAD: Brain-predicted age difference; GMV: Gray matter volume; WMV: White matter volume; CT: Cortical thickness.

### Predictive performance of brain age models

In the explorative binary logistic regression analyses, chronological age, CT-based brain age and brain-PAD were included as predictor variables. We found that pre-treatment estimated brain age was not a significant factor for clinical remission. As shown in Table 4, the score of CT-based brain-PAD was a significant predictor for the treatment responses to active rTMS, not sham rTMS at 3^rd^ week. We found that every additional year of pre-treatment CT-based brain-PAD decreased the odds of clinical remission by ∼25.7% in responders (OR = 0.743, Nagelkerke *R*^2^ = 0.392, *p* = .045) and by ∼39.5% in remitters (OR = 0.605, Nagelkerke *R*^2^ = 0.606, *p* = .022) in active rTMS group.

**Table 4.** Estimated effect sizes for the brain age models included in the predictive model of rTMS treatments (Responder).

	3^rd^ week (Post-treatment)				6^th^ week				12^th^ week (Endpoint)			
	OR	95% CI		*p* value	OR	95% CI		*p value*	OR	95% CI		*p value*
		Lower	Upper			Lower	Upper			Lower	Upper	
**Active rTMS group**												
CT-based brain age	0.868	0.647	1.164	.344	0.902	0.661	1.233	.519	0.952	0.737	1.228	.704
CT-based brain-PAD	0.743	0.556	0.994	**.045**	0.874	0.699	1.093	.237	0.808	0.638	1.024	.078
Global GMV	1	1	1	.975	1	1	1	.694	1	1	1	.262
Global WMV	1	1	1	.072	1	1	1	.289	1	1	1	.641
Mean CT	3.883	0.231	65.148	.346	5.947	0.219	161.301	.291	1.632	0.109	24.469	.723
**Sham rTMS group**												
CT-based brain age	0.827	0.615	1.112	.208	0.929	0.698	1.235	.611	0.838	0.627	1.119	.231
CT-based brain-PAD	0.924	0.764	1.117	.414	1.106	0.911	1.343	.308	1.071	0.905	1.265	.428
Global GMV	1	1	1	.409	1	1	1	.616	1	1	1	.674
Global WMV	1	1	1	.621	1	1	1	.581	1	1	1	.402
Mean CT	0.01	0	141.627	.156	0.01	0	135.807	.161	0.01	0	135.714	.178

Data are raw scores and presented as mean ± SD.

rTMS: Repetitive transcranial magnetic stimulation; OR: Odds Ratio; CI: Confidence Interval; Brain-PAD: Brain-predicted age difference; GMV: Gray matter volume; WMV: White matter volume; CT: Cortical thickness.

### Associations between brain age models and cortical excitability

In the active rTMS group, the responders showed significant RMT changes than non-responders at 3^rd^ week (RMT_23_: *t* = −2.472, *p* = .022), 6^th^ week (RMT_13_: *t* = −2.188, *p* = .04; RMT_23_: *t* = −3.102, *p* = .005) and 12^th^ week (RMT_23_: *t* = -2.472, *p* = .022) (Table 5). No RMT changes were found in the responders in the sham rTMS group.

**Table 5.** Changes of cortical excitability in the responders and remitters in two randomised groups.

Neuroplastic changes	Active rTMS group				Sham rTMS group			
	Non-responders	Responders	*t* value	*p* value	Non-responders	Responders	*t* value	*p* value
**3rd week**								
RMT12	2.75 ± 4.89	5.25 ± 6.65	−0.883	.387	0.09 ± 0.29	0.75 ± 0.96	−1.365	.263
RMT13	2.75 ± 4.89	8.25 ± 12.58	−1.544	.137	0.09 ± 0.29	0.01 ± 0.08	0.608	.549
RMT23	0.03 ± 0.16	3.72 ± 5.58	−2.472	.022	0.09 ± 0.29	−0.75 ± 0.96	1.535	.215
**6th week**								
RMT12	2.62 ± 4.81	7.01 ± 6.93	−1.408	.173	0.09 ± 0.29	1.01 ± 1.17	−1.565	.255
RMT13	2.62 ± 4.81	11.01 ± 13.85	−2.188	.041	0.09 ± 0.29	0.01 ± 0.08	0.525	.604
RMT23	0.03 ± 0.16	4.01 ± 6.93	−3.102	.005	0.01 ± 0.44	−1.01 ± 1.09	1.711	.223
**12th week**								
RMT12	2.75 ± 4.89	5.25 ± 6.65	−0.883	.387	0.05 ± 0.22	1.01 ± 0.82	−2.317	.101
RMT13	2.75 ± 4.89	8.25 ± 12.58	−1.544	.137	0.09 ± 0.29	0.01 ± 0.08	0.622	.541
RMT23	0.03 ± 0.16	3.01 ± 6.01	−2.472	.022	0.05 ± 0.38	−1.01 ± 0.82	2.514	.081

Data are raw scores and presented as mean ± SD.

rTMS: Repetitive transcranial magnetic stimulation; RMT: Resting motor threshold; RMT12: The baseline RMT minus the second measurement of RMT; RMT13: The baseline RMT minus the third measurement of RMT; RMT23: The second measurement of RMT minus the third measurement of RMT.

**Figure 4.**
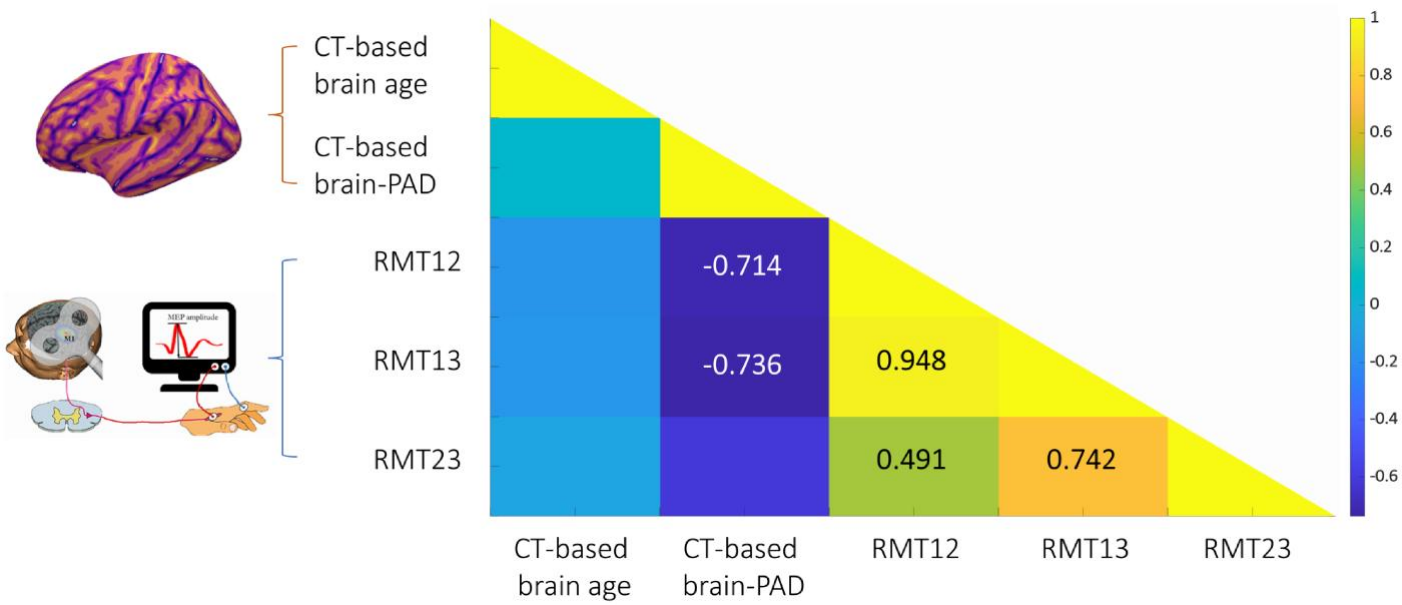
The correlation matrix of brain age model and the changes of cortical excitability. The lower scores of CT-based brain-PAD were related to enhanced cortical excitability. Abbreviations: CT: Cortical thickness; brain-PAD: Brain-predicted age difference.

Partial correlation analysis adjusted for chronological age and sex showed a negative association between the score of CT-based brain-PAD and the changes of RMT (RMT_12_: *r* = −0.714, *p* = .047; RMT_13_: *r* = −0.736, *p* = .038) in active rTMS group (Figure 4), indicating that the patients with a younger brain age exhibited more RMT changes than the patients with an older brain age. No significant association was found between the score of brain-PAD and RMT changes in the sham rTMS group.

### ROC analysis

**Figure 5.**
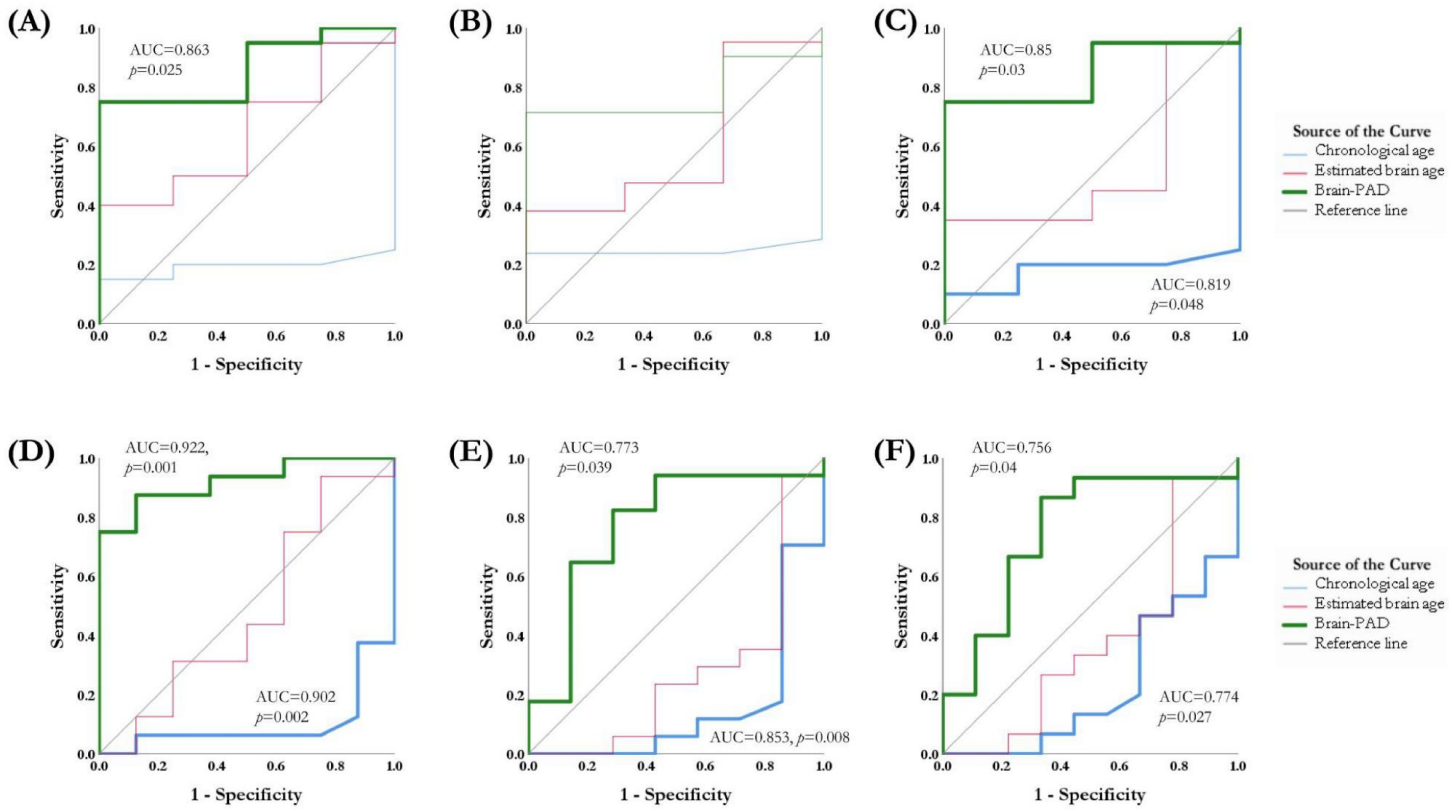
Receiver-operator characteristic (ROC) curves for the chronological age, estimated brain age and brain-PAD with differential values in the rTMS responders and remitters. The score of brain-PAD can differentiate the responders from the non-responders at the 3^rd^ week (A) and 12^th^ week (C). The chronological age and the score of CT-based brain-PAD can differentiate the remitters from the non-remitters at 3rd week (D), 6th week (E) and 12th week (F).

To classify the patients who responded well to rTMS, the values of the area under the ROC curve (AUC) were used to test the discriminative power of brain age models and cortical excitability. In the active rTMS group, the score of CT-based brain-PAD could differentiate the responders from the non-responders at 3^rd^ week (AUC = 0.863, 95%CI = 0.701–1, *p* = .025) (Figure 5A) and 12^th^ week (AUC = 0.85, 95%CI = 0.687–1, *p* = .03) (Figure 5C). The model reached 95.0% sensitivity and 75% specificity at the optimal cut-off point (brain-PAD = −4.86). As to the remitters, chronological age could differentiate the non-remitters from the remitters (AUC = 0.902, 95%CI = 0.771–1, *p* = .002), and CT-based brain-PAD score could differentiate the remitters from the non-remitters at 3^rd^ week (AUC = 0.922, 95%CI = 0.816–1, *p* = .001) (Figure 5D), 6^th^ week (AUC = 0.773, 95%CI = 0.545–1, *p* = .039) (Figure 5E) and 12^th^ week (AUC = 0.756, 95%CI = 0.542–1, *p* = .04) (Figure 5F). The model reached an average of 99.0% sensitivity and 62.5% specificity at the optimal cut-off point (brain-PAD = -1.87). The changes of RMT, chronological age and brain age models were unable to distinguish the responders and remitters in the sham rTMS group.

## Discussion

In this secondary analysis of a randomized clinical trial, we estimated the MRI-informed morphometric feature-based brain age model and their predictive capacities in the responses to rTMS in patients with co-occurring depression and cognitive impairments. Different from the discrepancies in chronological age, the responders and remitters who received active rTMS had a younger estimated brain age and a lower score of CT-based brain-PAD than the non-responders and non-remitters. Besides, the logistic regression analysis that evaluated the values of pre-treatment brain-PAD scores demonstrated substantially higher odds of better treatment outcomes (i.e., clinical remission) in those with a younger brain age. The results of this study provide an account of individual’s morphometric features as well as brain age models using structural MRI data in elderly patients who receive 10 Hz rTMS over the left DLPFC; the focus is on ascertaining the links between brain age models and treatment outcomes, and how the discrepancies in the treatment outcomes are related to cortical excitability.

### Brain age models and treatment outcomes

MRI-informed brain age model has been well investigated in clinical populations. Partially in line with published reports (Berk et al. 2010; Gaser et al. 2013; Wang et al. 2019; Karim et al. 2021; Ballester et al. 2022; Sone et al. 2022), increased estimated brain age was also found in our cases, indicating that the patients with depression and cognitive impairments had accelerated brain atrophy than the chronological age-matched normal controls. At the individual level, the differences between brain age and chronological age are related to the changes of cognitive statuses. For instance, increased brain-PAD score was strongly associated with the cognitive deterioration, suggesting that the individual with an older brain a has a higher risk of developing dementia (Gaser et al. 2013; Wang et al. 2019; Ballester et al. 2022). It is nevertheless important to acknowledge that even in elderly patients, accelerated brain ageing (i.e., older brain age) were found to contribute to severe depressive symptoms and poor clinical outcomes (Berk et al. 2010; Karim et al. 2021; Sone et al. 2022; Jha et al. 2023).

In the context of accelerated brain ageing, another important issue in the evaluation of predictive biomarkers in depressed patients would be addressed in this study. As mentioned, chronological age plays a critical role in the prediction of treatment responses and clinical outcomes at the group level (Figiel et al. 1998; Fabre et al. 2004; Lu et al. 2022). Still, whether depressed patients were younger or older, the prognostic usefulness of age remained unclear (Figiel et al. 1998; Fabre et al. 2004). Although we found that older chronological age was significantly correlated with the clinical remission of depressive symptoms in our primary results (Lu et al. 2022), when including brain-PAD as a covariate, the previously significant association between chronological age and better treatment outcomes disappeared. Thus, it is likely that the gap between estimated brain age and chronological age was related to better responses to the treatment. A recent clinical trial reported that lower baseline brain-PAD was associated with greater symptomatic improvements in young depressed patients who received the 8-week treatment of sertraline (Jha et al. 2023). Consistent with this study, the results of this secondary analysis also support the abovementioned assumption and show the robustness of the predictive values of CT-based brain-PAD over a relatively long-time frame (i.e., 3 months). Of note, to the best of our knowledge, this preliminary result is the first evidence of highlighting the specific brain feature (i.e., CT) in computing the brain age models and their predictive capacities in old depressed patients who received repeated rTMS treatment.

For a better understanding the discrepancy in chronological age and estimated brain age, the theory of neuroprogression might partially explain the mechanisms of accelerated brain ageing in elderly patients suffering from depression and comorbidities. Neuroprogression is characterised as a combination of morphometric changes (gray matter loss) and treatment nonresponsiveness that includes mild to moderate degenerative process, lower level of neural plasticity and neurogenesis in brain diseases, especially mood disorders (Moylan et al. 2013; Ruiz et al. 2018; Varghese et al. 2022). Recent studies found that the neuroprogressive process, exacerbating with the severity of depression, eventually leads to the deterioration or loss of neurocognitive functions (Han et al. 2021; Montejo et al. 2022), and progressive brain structural abnormality (Byers and Yaffe 2011). Neuroimaging studies applied to patients with depression and cognitive impairments have presented the morphometric changes of cortical regions that bring into evidence of different levels of reduced neural plasticity (Han et al. 2021). Among the features of neuroprogression, neural plasticity or the change of cortical excitability, is the most important one that may link neural mechanisms of rTMS and related beneficial effects (i.e., better treatment response). In support of this assumption, we found that the lower CT-based brain-PAD (i.e., younger brain), associated with a higher level of cortical excitability, can differentiate the responders from the nonresponders.

### Cortical excitability and its associations to brain age model

The inter-relationship of depression and comorbidities is complex, especially when the two co-occurring entities (i.e., depression and cognitive impairments) might be indistinguishable and complicating the statuses of brain ageing and neural plasticity at the individual level (Wu et al. 2021; Ferrarelli 2022). As a consequence, comorbidities lead to heterogeneity in brain features and cortical excitability, which accordingly increases the inter-individual variability in the treatment response to rTMS. The neuroplasticity hypothesis proposes that reduced neural plasticity is an underlying pathogenic mechanism in depression (Mesulam 1999) and neurodegenerative diseases (Ge et al. 2022). We observed that the enhancement of cortical excitability (i.e., decreased RMT) was presented in the responders who received active rTMS treatment. Interestingly, significant changes in RMT were found between the 10th to 15th session of rTMS treatment (i.e., RMT_23_). Although the treatment duration in our study (15 sessions) was less than the regular rTMS treatment in depressed patients (20 sessions), the rapid changes of cortical excitability in responders might echo a recent study that examined the predictive values of neuroplastic changes after a single session of rTMS (Ge et al. 2022). Thus, this interpretation of rTMS-induced RMT changes is aligned with the conventional use of TMS as a neuroplasticity probe and may be relevant to the severity of brain degeneration.

When linking RMT changes with the brain age model, we found that lower CT-based brain-PAD was significantly correlated with enhanced cortical excitability in the active rTMS group, indicating that the patients with a younger brain age exhibited more RMT changes than the patients with an older brain age. Indeed, a younger brain age is thought to reflect a delayed ageing process or a higher brain reserve, and possibly protects against pathological ageing. Besides, the presence of a younger brain age on better treatment outcomes could possibly indicate that this protective effect is closely relevant to an individual’s neuroplasticity or cortical excitability. Thus, differences in cortical excitability and brain age might provide a mechanistic explanation for the inter-individual variability in treatment responses to rTMS in elderly patients.

Focusing on brain age models, the predictive values of brain feature-specific brain age and brain-PAD on treatment outcomes were very limited, with only CT-based brain-PAD remaining as a predictor in our models. Instead of volume-based morphometric feature, the brain-PAD calculated based on cortical thickness turned out to be only associated with the statuses of responders and remitters in the active rTMS group, not in the sham group. The results highlight the brain-PAD’s potential utility in the pre-treatment evaluation of suitable participants (i.e., patient stratification). Notably, whether the brain age model could be used as predictive markers in other modalities of brain stimulation, such as transcranial current stimulation, or whether the re-organized neural network or altered cortical morphometry contributes to the treatment outcomes still needs to be in-depth investigated in future studies.

Nonetheless, from a clinical perspective, there might be two important implications of our findings that may inform the development of personalised brain-based treatments. First, brain age models are critical for recruiting suitable candidates for TMS treatment prior to the randomisation in clinical trials. For example, the patient with a younger brain age or a lower score of brain-PAD might be assigned to the subgroup receiving active TMS treatments. Second, after group assignment, MRI-informed brain features, and feature-specific brain age and the brain-PAD score could be used to construct the pre-treatment head model for optimising the stimulation target and treatment dosage at the individual level.

## Limitations

The findings of this study should be carefully interpreted in the context of limitations. Most notably, without detailed information of the subtypes of neurocognitive disorders, we are unable to evaluate the effectiveness of rTMS in disease-specific populations. In other words, subtypes of neurocognitive disorders might have been a source of variability confounding the results. For instance, individuals with NCD-AD and NCD-vascular may have similar clinical symptoms of depression and cognitive deficits, which are due to different aetiologies and neuropathologies. We considered this in our analyses by controlling the baseline clinical and morphometric features across the participants, including the scores of medial temporal atrophy (MTA) (i.e., AD-specific changes in the brain) and white matter hyperintensity (WMH) (i.e., vascular effects on the brain). We found no differences in the scores of MTA and WMH between non-responder/responder and non-remitter/remitter in active and sham rTMS groups (Table S3).

Another important limitation is the total TMS dose (i.e., duration of treatment) applied in this study. Compared to the standard-of-care FDA-approved rTMS protocol for treating major depressive disorder, our protocol only applied half of the total number of TMS pulses (i.e., 1500 pulses). Although our main purpose was to examine the rapid antidepressant and cognitive effects of rTMS in NCD patients, the shortened duration of treatment and reduced TMS pulses may weaken the therapeutic effect and neuroplasticity induced by active rTMS. Moreover, the responders who received fifteen sessions of rTMS treatment in our study might be ‘faster responder’, which might limit the generalisation of our findings in brain age and treatment outcomes.

In addition, it is also important to note the use of medication in elderly patients. It is difficult to control and quantify the effects of medication on neuroplastic potential and brain age models and that the participants routinely take to treat their chronic diseases. Although no differences in the medication regimes and doses were found in the nonresponders/responders and nonremitters/remitters, there may be potential influence of medications and chronic diseases on the response to rTMS that cannot be evaluated separately. On the other hand, this situation reflects real-world practice in hospitals, where patients typically maintain a regular medication regimen rather than discontinue pharmacological treatment.

## Conclusion

In summary, our findings may provide the basis for a prospective randomised clinical trial using neuroimaging and physiological biomarkers. From a clinical perspective, the present findings indicated that it may be possible to prospectively identify the candidates who are more likely to benefit from active rTMS treatment for depression. The responders and remitters had younger brain age (i.e., more brain reserve) and better neuroplastic potential (i.e., cortical excitability). Pre-treatment brain age models in patients with depression and neurocognitive disorder are relevant to the inter-individual variability in treatment outcomes, which may index the macro-level morphometric features and inform predictive biomarkers.

## Supplementary Material

Supplemental Material

Supplemental Material

## Data Availability

The source code and pre-trained models of brain age metrics have been uploaded to our GitHub page (https://github.com/hannabrainscience/Brain-age-prediction).

## Data availability statement

Raw and processed imaging data under the General Data Protection Regulations (GDPR) may be available for research collaboration purpose upon reasonable request to the corresponding authors (Hanna Lu and Linda Chiu Wa Lam) and will require the completion of a data processing agreement.
