# Supplementary material for: Predictive values of pre-treatment brain age models to rTMS effects in neurocognitive disorder with depression: Secondary analysis of a randomised sham-controlled clinical trial
Source: Dialogues Clin Neurosci. 2024 Jul 4;26(1):38–52. doi: 10.1080/19585969.2024.2373075 (PMC11225634; doi:10.1080/19585969.2024.2373075)
Supplement: Supplemental Material [file TDCN_A_2373075_SM5777.docx]

| Left GMV (34) | Right GMV (34) | Left GMT (33) | Right GMT (33) |
| --- | --- | --- | --- |
| L_SFG_GMV | R_SFG_GMV | L_SFG_GMT | R_SFG_GMT |
| L_MFG_GMV | R_MFG_GMV | L_MFG_GMT | R_MFG_GMT |
| L_OplFG_GMV | R_OplFG_GMV | L_OplFG_GMT | R_OplFG_GMT |
| L_TrIFG_GMV | R_TrIFG_GMV | L_TrIFG_GMT | R_TrIFG_GMT |
| L_OrlFG_GMV | R_OrlFG_GMV | L_OrlFG_GMT | R_OrlFG_GMT |
| L_PrG_GMV | R_PrG_GMV | L_PrG_GMT | R_PrG_GMT |
| L_TFG_GMV | R_TFG_GMV | L_TFG_GMT | R_TFG_GMT |
| L_GRe_GMV | R_GRe_GMV | L_GRe_GMT | R_GRe_GMT |
| L_MOrG_GMV | R_MOrG_GMV | L_MOrG_GMT | R_MOrG_GMT |
| L_AOrG_GMV | R_AOrG_GMV | L_AOrG_GMT | R_AOrG_GMT |
| L_POrG_GMV | R_POrG_GMV | L_POrG_GMT | R_POrG_GMT |
| L_LOrG_GMV | R_LOrG_GMV | L_LOrG_GMT | R_LOrG_GMT |
| L_ParacentralLobule_GMV | R_ParacentralLobule_GMV | L_ParacentralLobule_GMT | R_ParacentralLobule_GMT |
| L_CgG_GMV | R_CgG_GMV | L_CgG_GMT | R_CgG_GMT |
| L_SCA_GMV | R_SCA_GMV | L_SCA_GMT | R_SCA_GMT |
| L_PoG_GMV | R_PoG_GMV | L_PoG_GMT | R_PoG_GMT |
| L_SMG_GMV | R_SMG_GMV | L_SMG_GMT | R_SMG_GMT |
| L_AnG_GMV | R_AnG_GMV | L_AnG_GMT | R_AnG_GMT |
| L_SPL_GMV | R_SPL_GMV | L_SPL_GMT | R_SPL_GMT |
| L_Pcu_GMV | R_Pcu_GMV | L_Pcu_GMT | R_Pcu_GMT |
| L_tmp_GMV | R_tmp_GMV | L_tmp_GMT | R_tmp_GMT |
| L_STG_GMV | R_STG_GMV | L_STG_GMT | R_STG_GMT |
| L_TTG_GMV | R_TTG_GMV | L_TTG_GMT | R_TTG_GMT |
| L_MTG_GMV | R_MTG_GMV | L_MTG_GMT | R_MTG_GMT |
| L_ITG_GMV | R_ITG_GMV | L_ITG_GMT | R_ITG_GMT |
| L_FuG_GMV | R_FuG_GMV | L_FuG_GMT | R_FuG_GMT |
| L_PHG_GMV | R_PHG_GMV | L_PHG_GMT | R_PHG_GMT |
| L_HiF_GMV | R_HiF_GMV | / | / |
| L_SOG_GMV | R_SOG_GMV | L_SOG_GMT | R_SOG_GMT |
| L_MOcG_GMV | R_MOcG_GMV | L_MOcG_GMT | R_MOcG_GMT |
| L_IOG_GMV | R_IOG_GMV | L_IOG_GMT | R_IOG_GMT |
| L_LiG_GMV | R_LiG_GMV | L_LiG_GMT | R_LiG_GMT |
| L_Cun_GMV | R_Cun_GMV | L_Cun_GMT | R_Cun_GMT |
| L_Ins_GMV | R_Ins_GMV | L_Ins_GMT | R_Ins_GMT |

Supplementary Table 1. Independent variables for the prediction model of brain age

Abbreviations: SFG: superior frontal gyrus; MFG: middle frontal gyrus; OplFG: pars opercularis; TrIFG: pars triangularis; OrlFG: pars orbitalis; PrG: pre-central gyrus; TFG: transvers frontal gyrus; GRe: gyrus rectus; MOrG: middle orbito-frontal gyrus; AOrG: anterior orbito-frontal gyrus; POrG: posterior orbito-frontal gyrus; LOrG: lateral orbitofrontal gyrus; ParacentralLobule: paracentral lobule; CgG: cingulate gyrus; SCA: subcallosal gyru; PoG: post-central gyrus; SMG: supramarginal gyrus; AnG: angular gyrus; SPL: superior parietal gyrus; Pcu: pre-cuneus; tmp: temporal pole; STG: superior temporal gyrus; TTG: transverse temporal gyrus; MTG: middle temporal gyrus; ITG: inferior temporal gyrus; FuG: fusiforme gyrus; PHG: parahippocampal gyrus; HiF: hippocampus; SOG; superior occipital gyrus; MOcG: middle occipital gyrus; IOG: inferior occipital gyrus; LiG: lingual gyrus; Cun: cuneus; Ins: Insula.
