# Supplementary figures and images for: Predictive values of pre-treatment brain age models to rTMS effects in neurocognitive disorder with depression: Secondary analysis of a randomised sham-controlled clinical trial
Source: Dialogues Clin Neurosci. 2024 Jul 4;26(1):38–52. doi: 10.1080/19585969.2024.2373075 (PMC11225634; doi:10.1080/19585969.2024.2373075)

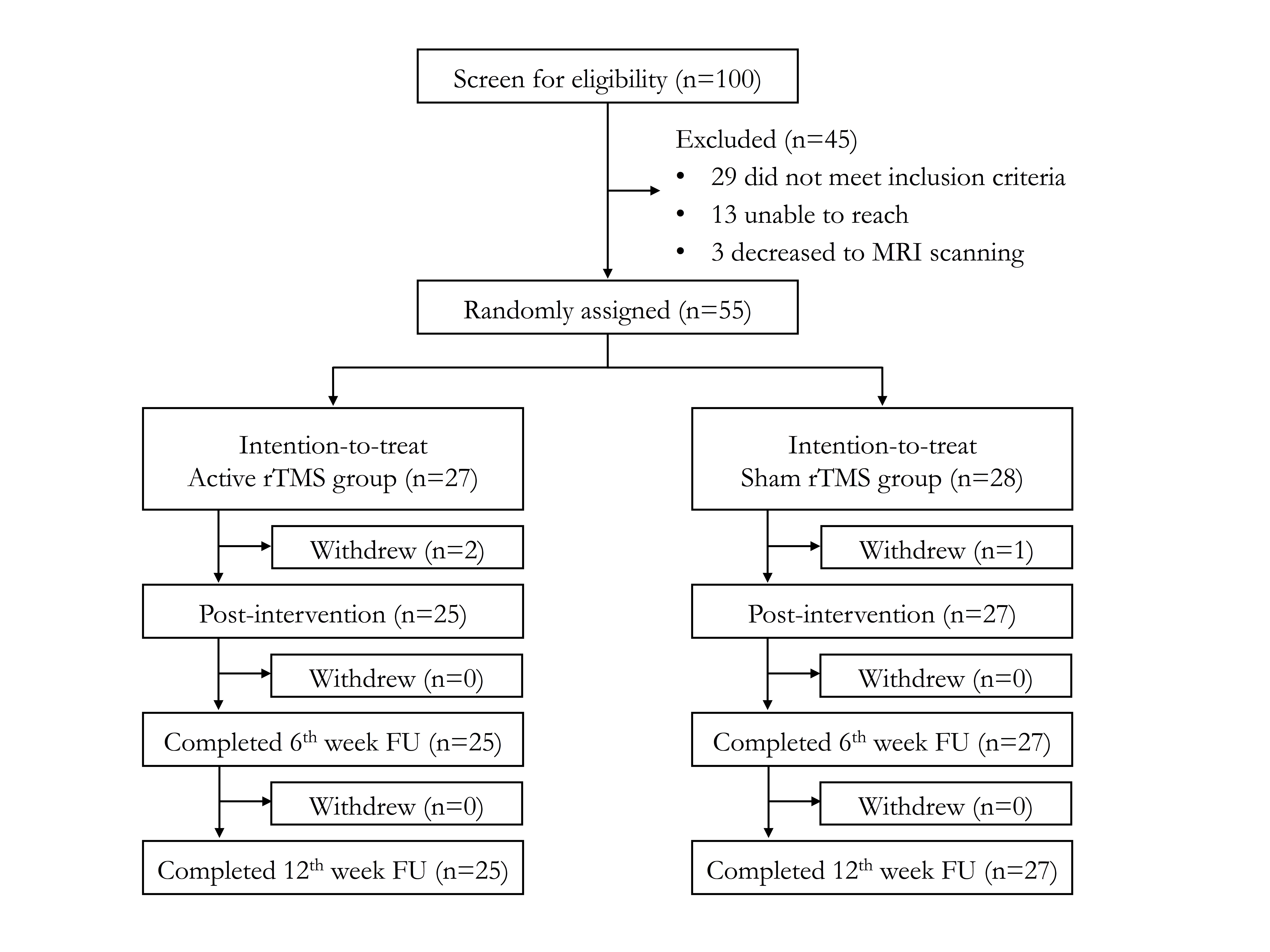

Supplement: Supplemental Material [file TDCN_A_2373075_SM5776.jpg]
